# Effects of Crystal Orientation and External Stress on the Static Recrystallization Behavior of an Ni-Based Single-Crystal Superalloy

**DOI:** 10.3390/ma17133123

**Published:** 2024-06-26

**Authors:** Fuze Xu, Yongcheng Lin, Dexin Ma, Wei Xiong, Daoguang He, Guan Liu, Yunxing Zhao

**Affiliations:** 1 School of Mechanical and Electrical Engineering, Central South University, Changsha 410083, China; xufuze12@csu.edu.cn (F.X.); 233712182@csu.edu.cn (G.L.); 2 Shenzhen Wedge Central South Research Institute Co., Ltd., Shenzhen 518045, China; madexin@csu.edu.cn (D.M.); 213711012@csu.edu.cn (W.X.); c2652104270@163.com (Y.Z.); 3 Powder Metallurgy Research Institute, Central South University, Changsha 410083, China

**Keywords:** single-crystal superalloy, crystal orientation, solution heat treatment, static recrystallization

## Abstract

The deformation mechanism and static recrystallization (SRX) behavior of an Ni-based single-crystal superalloy are investigated. Indentation tests were performed to investigate the effects of crystal orientation and external stress on SRX behavior. Following solution heat treatment, the depth of the SRX layer below the indentation increases with a deviation angle (β) from the [001] orientation. The slip analysis indicates that an increased deviation angle leads to an increase in the resolved shear stress on the slip plane and a decrease in the number of active slip systems. In addition, the variation pattern of the SRX layer depth with the deviation angle is consistent for different external stresses. The depth of the SRX layer also increases with external stress. The coarse γ′ phases and residual γ/γ′ eutectics obviously enhance the pinning effects on the expansion of recrystallized grain boundaries, resulting in slower growth rates of the recrystallized grains in interdendritic regions than those in dendrite core regions.

## 1. Introduction

Superalloys are extensively used in the manufacture of aeroengine turbine blades owing to their excellent high-temperature oxidation resistance and creep properties [1,2]. The excellent mechanical properties of single-crystal (SC) superalloy castings at elevated temperatures may be attributed to the elimination of grain boundaries [3,4,5]. Static recrystallization (SRX) [6,7,8] usually induces grain boundaries in alloys, such as SC superalloy castings, which significantly affects the creep and fatigue properties of alloy components [9]. After directional solidification (DS), the casting mold typically undergoes processes such as shell removal and cutting. Castings are susceptible to local stress concentrations caused by mechanical impacts during transportation, shell removal, and mechanical processing. This can lead to SRX in the affected areas during subsequent heat treatment or service [10,11].

Previous studies have primarily focused on the nucleation and growth mechanisms of SRX in SC superalloys [12,13,14,15,16,17]. Typically, experimental methods, such as indentation testing [13,18,19], shot peening [20,21], grit blasting [22,23], and hot compression [24,25], are employed to pre-deform the specimen. The evolution of SRX in the plastic deformation zone of the specimen was studied through heat treatment experiments. Recently, the correlation between plastic deformation and SRX has been analyzed using numerical simulations [26,27]. It has been found that alloying elements, such as Al, W, and Re, as well as carbides, have a significant impact on the formation and growth of SRX in SC superalloys [28,29,30,31]. Additionally, research has shown that, in some SC superalloys, the undissolved γ′ phase may hinder the migration of SRX grain boundaries and assist in the nucleation of SRX [32]. Furthermore, some researchers [33,34,35,36,37] have found that crystal orientation significantly affects the microstructural features and fatigue properties of SC superalloys. Gao et al. [38] investigated how deviation angles of 3°, 8°, and 13° from the [001] orientation affect the tensile properties and deformation behavior of an Ni-based SC superalloy at room temperature. Their findings reveal that strength decreases and ductility increases as the deviation angle widens within the [001] orientation. Burgel et al. [24] investigated the SRX behavior of the CMSX-6 alloy by indentation tests and found that SRX areas along the [100] and [110] crystal orientations were different. Xie et al. [19] studied the orientation dependence of the deformation and SRX in an Ni-based SC superalloy indented on the (100) and (110) planes. Qin et al. [39] investigated the stress and orientation dependence of oxidation-induced dynamic recrystallization (DRX) in an Ni-based SC superalloy during high-temperature creep. More recently, Jiao et al. [40] found higher crystal distortion and obvious SRX behavior in the [111] orientation than in the [110] and [112] orientations of the Mo-3Nb SC superalloy. To gain a deeper insight into the effect of crystal orientation on SRX, Wei et al. [41] investigated the effect of primary and secondary orientations on the cyclic plastic behavior and fatigue properties of recrystallized SC superalloys. They found that primary orientation significantly influences the accumulated plastic shear strain in the recrystallized SC superalloy. To efficiently manage the crystal orientation of single-crystal turbine blades, Qin et al. [42] have developed a novel high-capacity mold for producing single-crystal blades with consistent orientation.

Nevertheless, the predominant emphasis of these investigations has been on the comparative analysis of specific-crystal orientations, such as [001,110], and [111]. In industrial applications, the deviation angle between the primary axial and the [001] orientation of the casting should not exceed 15° or even be smaller. However, SC castings may experience dents during shelling, cutting, and transportation due to collisions. The deformation at the dents can easily induce SRX during subsequent heat treatment. The angle between the normal direction of the dent and the [001] orientation of the casting typically varies significantly, and the extent of its deformation is uncertain. Hence, this study employs various stress indentation tests to investigate the impact of changes in orientation on the process of SRX. This investigation carries substantial engineering importance in evaluating the extent of SRX induced by heat treatment subsequent to casting collision. Moreover, this article also offers an in-depth analysis of the deformation mechanism exhibited by specimens with different crystal orientations in response to indentation.

## 2. Materials and Experimental Procedures

**Figure 1:**
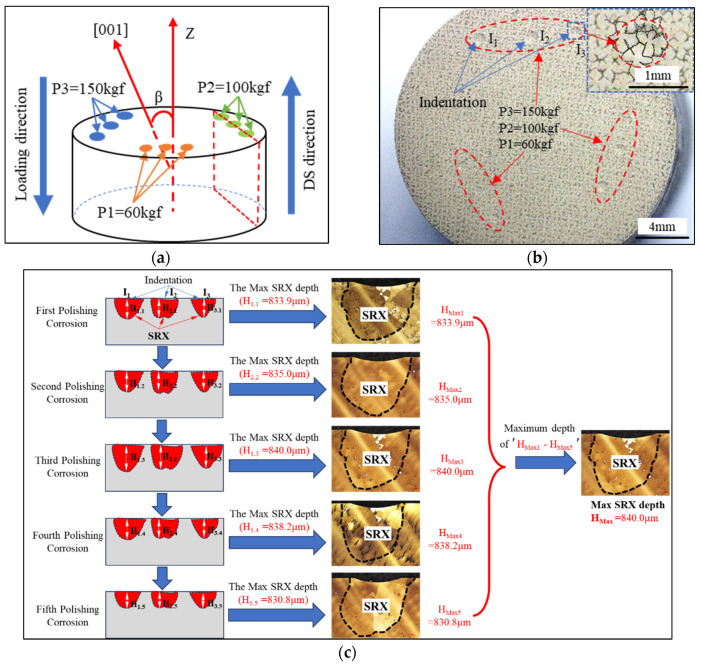
(**a**) Schematic diagram of indentation, (**b**) surface metallograph of sample after indentation, and (**c**) schematic diagram of the method for obtaining the maximum depth of SRX in samples.

The experimental material was a second-generation SC superalloy, DD5, the nominal composition of which is listed in Table 1. The SC rods were produced using the screw crystal selection method in an industrial vacuum Bridgman furnace (ALD vacuum precision casting furnace). The preheating temperature of the mold shell was 1550 °C, the pulling rate of directional solidification was 3 mm/min, and all SC rods were prepared under the same solidification conditions. The diameter and length of the SC rods were 15 and 170 mm, respectively. The orientations of rods were measured by the X-ray Laue method, and six SC rods with a deviation angle (β) from 4.7° to 48.4° were used. The symbol β represents the deviation angle between the [001] orientation and the primary axial (also the load direction and the DS direction) (Figure 1a). The corresponding rods are labeled A–F, as shown in Table 2.

The cylindrical samples, with dimensions of Φ15 mm × 8 mm, were sliced perpendicular to the axis of the test rods (aligned with the DS direction) using electrical-discharge machines (EDMs). The cut surfaces of the samples were pre-deformed at room temperature using a hardness tester (model HRD150) with a spherical indenter (diameter, 0.5 mm) (Figure 1a). External indentation loads of 60, 100, and 150 kgf were applied and held for 10 s.

As illustrated in the indentation diagram in Figure 1a, the applied loads are labeled as P_1_, P_2_, and P_3_, corresponding to 60, 100, and 150 kgf. Each load was applied three times. The indentation load positions were arranged in a single row, facilitating the statistical analysis and observation of the SRX in the longitudinal section. Figure 1b shows an image of the indentation of the sample, where I_1_–I_3_ represent three indents with the same applied load. Each indentation area in the sample contains multiple dendrites (2 to 4), as shown in the local magnification in Figure 1b. All the sample sizes meet the requirements of current standards. Following the deformation, half of the distorted samples were preserved in their initial as-cast condition. The remaining half underwent solution heat treatment using a 15.0 VPT-4022/24HVIQ furnace under vacuum conditions. The solution heat treatment procedure consists of three insulation stages: 1280 °C/2 h + 1290 °C/1 h + 1300 °C/2 h, followed by cooling with argon gas. The heating rate during the process from room temperature to 1150 °C was 10 °C/min, while the heating rate above 1150 °C was 3 °C/min.

To observe the dislocation and slip phenomenon, as well as SRX behavior below the indentation, some as-cast samples and all heat-treated samples were cut along the mid-section perpendicular to the indentation surface by EDM (shown as the dashed box in Figure 1a). For the heat-treated indentation samples, five SRX morphologies were observed near the bottom of each indentation after five consecutive rounds of polishing and etching (FeCl_3_:HCl:H_2_O = 1:1:2), five different SRX morphologies were observed near the bottom of each indentation, and a total of 15 SRX morphologies was obtained. The image with the largest SRX depth was selected for further tissue observation and statistical analyses. Figure 1c shows a schematic of the process for achieving the maximum SRX depth, where I_1_, I_2_, and I_3_ are three indentations under the same load. The red area below the indentations represents the schematic of the SRX profile.

The macroscopic morphology of the SRX grains was observed using a NIKON MM-400 optical microscope (OM), and the microstructures were analyzed using an FEI Quanta 650 scanning electron microscope (SEM). The areas of interest were finely polished for electron backscatter diffraction (EBSD) observation; the model was JEOL-7001F1 FE-SEM. The EBSD sample was taken at a voltage of 25 kV, a current of 11 nA, and a step size of 1.6 μm. Additionally, the EBSD data were processed using OIM (8.5.1002 X64) to analyze the local orientation differences of the cast and heat-treated states under various deformations.

## 3. Results and Discussion

### 3.1. Effects of Crystal Orientation on Recrystallization Behavior

#### 3.1.1. As-Cast Dendrite Morphology and Microstructure Characteristics

**Figure 2:**
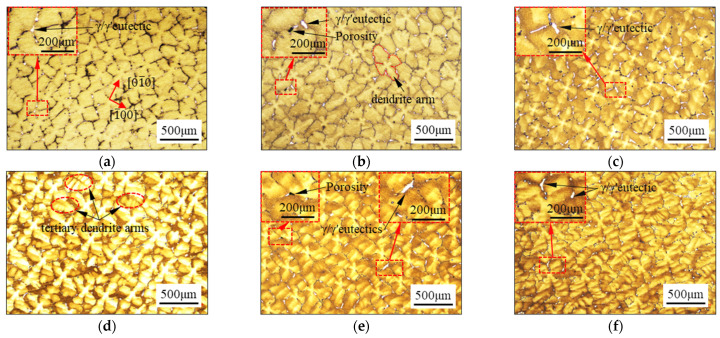
As-cast microstructure on the cross-sections with the β of: (**a**) 4.7°, (**b**) 14.7°, (**c**) 27.8°, (**d**) 36.8°, (**e**) 39.8°, and (**f**) 48.4°.

Figure 2 illustrates the as-cast dendrite morphology and microstructural characteristics of different crystal samples at the cross-section (vertical indentation direction). In Figure 2a–c, the primary dendrite morphology on the cross-section exhibits a typical symmetrical ‘cross’-pattern structure with 4.7° ≤ β ≤ 27.8°. While the value of β is 36.8°, the primary dendrite has an asymmetric ‘cross’-pattern structure, and the tertiary dendrite arms can be found, which are marked by the red dotted lines in Figure 2d. However, when the value of β is increased to 39.8° and 48.4°, the cross-section of dendrite arm morphology is significantly deflected, and no symmetrical cross-pattern structures are observed. In particular, when the value of β is 48.4°, the primary dendrite morphology becomes messy and interspersed with some secondary and tertiary dendrites. The variations in the dendrite morphology in the cross-section can be interpreted as being related to the various angles between the cut section and the [001] orientation [44].

It is evident that white-striped γ/γ′ eutectics exist in the interdendritic regions on the cross-section of the samples, regardless of their crystal orientation. Additionally, small micropores have been observed in close proximity to certain γ/γ′ eutectics, as shown by the black spots indicated by the arrows in Figure 2b,e. However, despite the changes in crystal orientation, no noticeable variation is observed in the volume fraction of γ/γ′ eutectics and micropores on the observed surface in the cross-sections.

#### 3.1.2. Recrystallization Behavior at Different Crystal Orientations

**Figure 3:**
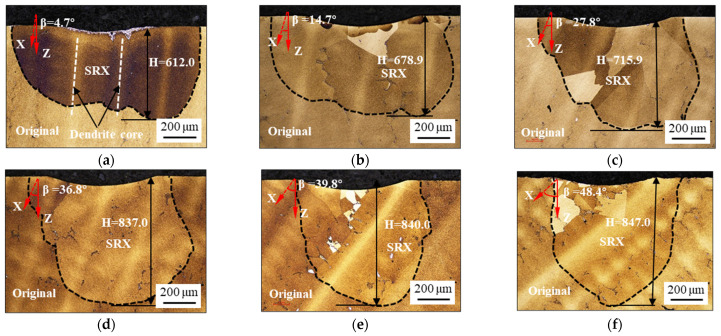
OM maps of SRX microstructure below the indentation at the load of 150 kgf with β values of: (**a**) 4.7°, (**b**) 14.7°, (**c**) 27.8°, (**d**) 36.8°, (**e**) 39.8°, and (**f**) 48.4°.

The characteristics of the SRX grains in the samples heat-treated at a load of 150 kgf are shown in Figure 3. A reference coordinate system, composed of the indentation direction and [001] crystal orientation, is added. In this reference coordinate system, the X-axis represents the [001] orientation, the Z-axis indicates the direction of the loaded indentation, and the angle between the X-axis and the Z-axis is denoted as β. A vertical load pressed a shallow pit with a depth of several microns onto the upper surface of each sample. Roughly hemispherical recrystallized grains exist with a depth of several-hundred microns below the shallow pit. Semi-elliptical or semi-circular SRX layers appeared in one or more grains. The SRX outline is delineated with black dashed lines in the figure, and the maximum depth of the SRX layer (H) was also evaluated. When β is 4.7° (Figure 3a), the value of H is approximately 612.0 μm along the indentation direction. When the value of β is 48.4° (Figure 3f), it is approximately 847.0 μm. The value of H increases with an increase in the value of β. As shown in Figure 3, the larger the value of β, the more pronounced the SRX behavior. Notably, the center of the indentation (directly below the indentation, as well as the position indicated by the arrow in Figure 3a) exists in the interdendritic region, while the maximum SRX appears on both sides of the indentation center (Figure 3a). This indicates that the degree of SRX in the dendrite core region is greater than that in the interdendritic region. Detailed discussions of the SRX mechanisms are provided in the following sections.

**Figure 4:**
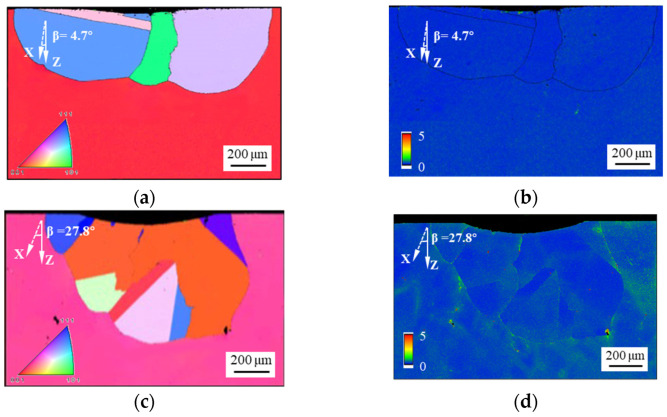

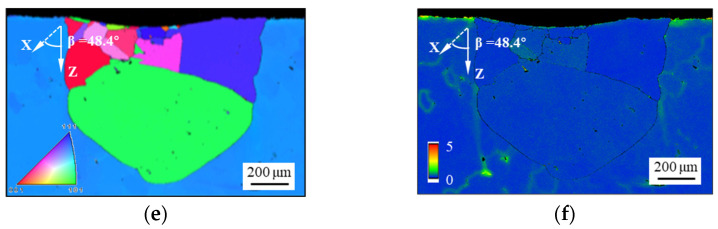
IPF and KAM maps of the SRX areas at 150 kgf indented samples with the following values of β: (**a**,**b**) 4.7°; (**c**,**d**) 27.8°; (**e**,**f**) 48.4°.

Figure 4 depicts the corresponding inverse pole figure (IPF) and kernel average misorientation (KAM) maps of SRX areas at an indentation load of 150 kgf and β values of 4.7°, 27.8°, and 48.4°. When the value of β is 4.7°, larger grains are formed after the indentation, and the plastic strain energy within the SRX region has been effectively released. However, small grains are observed at the bottom of the indention when the values of β are 27.8° and 48.4°, which implies a higher nucleation rate in these areas [26]. The number of SRX grains increases with increasing β, while the average size of SRX grains decreases. This is because the sample with a small orientation has almost completed the coarsening and growth of the recrystallized grains. The recrystallized grain boundaries and the residual γ/γ′ eutectics retain the unreleased energy that was stored during deformation, as illustrated in Figure 4d,f. Therefore, the growth and coarsening processes of the SRX grains are not yet complete, and SRX can proceed further. However, as mentioned above, no significant difference exists in the γ/γ′ eutectics and microporosity volume fraction of samples with different orientations in the cross- and longitudinal sections. Therefore, the variation in SRX depth is not primarily attributable to the pinning effect of γ/γ′ eutectics on SRX, but rather to the disparity in crystal orientation. The reason for this is discussed in the following paragraphs.

**Figure 5:**
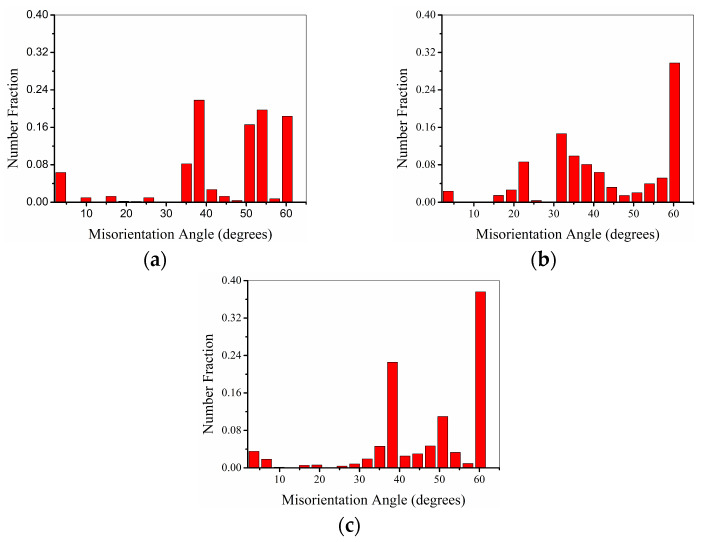
Angular misorientation distribution of SRX grains relative to SC matrix after heat treatment of samples with the following values of β: (**a**) 4.7°, (**b**) 27.8°, and (**c**) 48.4°.

The distribution of the orientation difference between SRX grains and the matrix below the indentation of samples with various crystal orientations after solution heat treatment is shown in Figure 5. The results indicate that the orientation difference between the SRX grains at the indentation and the matrix is mainly greater than 30 degrees. The number of SRX grains with larger orientation differences increases as the β value increases, but the growth is limited. When the β value is 4.7°, approximately 89.9% of the total number of SRX grains exhibit a crystal orientation difference greater than 30°. As the β value increases to 48.4°, the number of SRX grains with a crystal orientation difference greater than 30° is approximately 92.1% of the total number of SRX grains. The number of grains with SRX crystals showing an orientation difference of ≥60° varied significantly with the β-value. The percentages of SRX grains at the indentation of the specimens with β-values of 4.7°, 27.8°, and 48.4° in the total number of SRX grains were 18.4%, 29.7%, and 37.6%, respectively. The larger the misorientation, the easier the growth and coarsening of the SRX grains [42].

#### 3.1.3. Dislocation Slip Behaviors and Mechanisms in Various Crystal Orientations

(1) Dislocation slip behaviors in various crystal orientations

**Figure 6:**
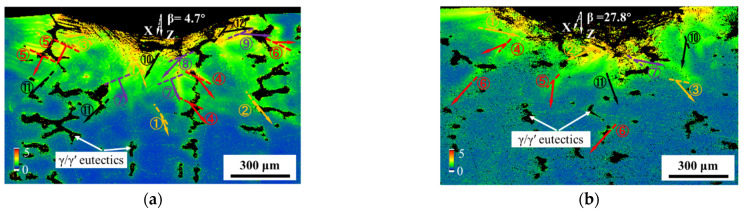

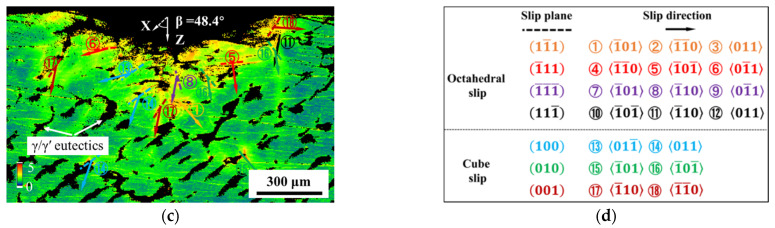
KAM maps annotated with slip systems of as-cast samples with β values of: (**a**) 4.7°, (**b**) 27.8°, and (**c**) 48.4°at the load of 150 kgf, and (**d**) corresponding slip system in (**a**–**c**).

Figure 6a–c show the KAM plots of the longitudinal section in the indentation center of cast samples with β values of 4.7°, 27.8°, and 48.4°. The indentation load was set to 150 kgf. In Figure 6a, the black discrete blocks and continuous bands in the KAM maps are γ/γ′ eutectics, as indicated by the white arrows. The orientations of the various dislocation slip systems were analyzed using the MTEX toolbox based on MATLAB (R2018b) analysis software [45]. The arrows in Figure 6a–c indicate the direction of the dislocation slip, and the dashed lines show the dislocation slip surface traces. The octahedral slip systems, as well as the cubic slip systems, are labeled in Figure 6d.

As a face-centered cubic crystal, the octahedral slip and cubic slip are the main slip deformation forms. Accordingly, they are also taken into account in most crystal plasticity models [46]. In this research, Schmid factors for the 12 octahedral slip systems and 6 cubic slip systems with β values of 4.7°, 27.8°, and 48.4°are listed in Table 3 and Table 4. Octahedral slip systems with Schmid factors below 0.15 are not labeled when the values of β are 27.8° and 48.4°. The results show that the highest misorientation occurs near the indenter, and the KAM gradually decreases with increasing distance from the indentation, which is consistent with the SRX distribution shown in Figure 3. At a depth of approximately 300 µm from the indentation, the misorientation is less than 0.5°, and the highest misorientation can only be found in the area around the local γ/γ′ eutectics. A significant horizontal KAM gradient can be seen on the right side of the 4.7° indentation, which agrees well with the slip direction of the three slip systems (⑥, ⑨, and ⑫), as indicated in Figure 6a. However, in Table 3, it can be found that the Schmid factors of slip systems ⑥, ⑨, and ⑫ are actually very small, which may be initiated by the stress component in the horizontal direction generated by the indenter pressing into the alloy. For the sample with a β of 4.7°, the traces of four slip faces with stronger interactions on the multiple slip faces are visible immediately beneath the indentation. The number of slip systems with a Schmid factor greater than 0.2 is lower for the sample with a β of 27.8°. The interaction of multiple slip systems below the indentation is noticeably weaker. In addition, the extension depth of a single slip system for the sample with a β of 27.8° is deeper than that with a β of 4.7° (slip system ⑥, Figure 6b).

The sample with a β of 48.4° has fewer traces of significant octahedral slip systems compared with the samples with β values of 4.7° and 27.8°. There are only four slip systems with Schmid factors higher than 0.2, which is related to the fact that the β of 48.4° is oriented closer to the [111] orientation. The Schmid factor of a cubic slip system is generally higher when the stress direction is closer to the [111] orientation [47]. Therefore, there are more cubic slip systems in Figure 6c. A high-stress concentration phenomenon is also observed at the γ/γ′ eutectics at an indentation depth of approximately 500 µm in the slip systems of ⑭ and ⑮. This indicates that the expansion depth of the compressive stress in the sample with a β of 48.4° is larger.

In this study, a spherical indenter was used to apply load to samples in a specific orientation. The force between the indenter and sample was directed perpendicular to the tangent at the contact point, causing a change in the stress direction in different indentation areas. This results in nonuniform stresses on the sample as localized deformation and the generation of geometrically necessary dislocations occur. The indentation depth plays a significant role in the variation in stress. This change in the force direction also triggers the activation of the slip system at different locations. Traces of the activity of three slip systems ⑥, ⑨, and ⑫ can be observed at the upper right of the sample with a β of 4.7° (Figure 6a). Because the Schmid factors of the three slip systems were less than 0.25, they were not preferentially activated under the vertical downward force. The slip traces shown in Figure 6a demonstrate the presence of horizontally oriented stresses that activate the slip system in these areas. The slip direction is consistent with the force in the horizontal direction. This indicates that the depth of SRX below the indentation is related to the magnitude of the force in the vertical direction. Schmid’s law is expressed as follows:

(1) τ=σ⋅m

where *τ* is the resolved shear stress (RSS) on the slip plane, *σ* is the stress on the sample, and *m* is the Schmid factor. In the depth direction of the indentation, the RSS on the slip plane is positively correlated with the Schmid factor, as listed in Table 3. The Schmid factors on the (100) and (010) slip planes of the sample with a β of 48.4° are 0.47 and 0.45, respectively. This indicates that the same force applied to the alloy produces a greater RSS and depth of extension below the indentation (Figure 6c). In addition, after heat treatment, the angle between the grain boundaries of the recrystallized grain for the sample with a β of 48.4° is essentially the same as that of the two {100} crystal planes. This indicates that the final recrystallized grain boundaries are formed by upward slip bands along the [100] orientation. Previous studies have shown that changes in the orientation of the stress axis can activate different slip systems [48,49,50]. Macroscopic cubic slip traces are present in the [111] samples, and such macroscopic slip traces result from the alternating sliding of dislocations on two neighboring octahedral slip planes [51,52]. This is consistent with the cubic slip traces observed in this study.

In addition to the differences in the magnitude of the RSS in samples with different orientations, the number of activated slip systems influences the SRX depth [26]. For the sample with a β of 4.7°, the Schmid factor of the eight slip systems is approximately 0.4, which indicates that cross-slip on different slip planes results in more uniform deformation on the lower side of the indentation. As the orientation of the samples changed from near [001] to [111], the depth of the SRX gradually increased. When the load is applied along the β direction, the number of activated slip systems (Ass) can be estimated using the Schmid factor. At room temperature, the primary deformation mechanism in SC superalloys is characterized by the shearing deformation of dislocations along the {111} <110> slip systems [53,54]. Continuous dislocation sliding on the slip plane produces a slip band [55]. The slip bands formed a misorientation gradient distribution and extended in a certain direction in the KAM maps (Figure 6). Furthermore, stronger interactions between multiple slip planes can make it difficult for deformation on the slip plane to extend downward. Therefore, as β increases, the RSS on the slip planes rises and the interaction of the slip surface weakens, which ultimately leads to an increase in the depth of the deformation region and SRX after heat treatment.

(2) Dislocation slip mechanisms in various crystal orientations

As mentioned above, the depth of the SRX below the indentation increased with the deviation of the orientation from the [001] orientation; this pertains to the mechanisms of deformation exhibited by various orientations. The deformation of a crystalline material under an external load consists of elastic and plastic deformation, as proposed by Hill [56], Asaro [57,58], and Pierce [59]. The dislocation movement in the crystal causes the lattice to deform, and the total deformation gradient *F* is given by

(2) $$F=F^{e}\cdot F^{p}$$

where *F^e^* is the elastic deformation, and *F^P^* is the plastic deformation. The rate of change of *F^P^* is related to the shearing rate, ${\dot{\gamma }}^{\left(a\right)}$, of the α slip system as follow:

(3) $${\dot{F}}^{p}\cdot F^{p-1}=\sum_{a}{\dot{\gamma }}^{\left(a\right)}s^{\left(a\right)}m^{\left(a\right)}$$

The rate of shearing, denoted as ${\dot{\gamma }}^{\left(a\right)}$, for the α slip system can be described as a function of the resolved shear stress, $\tau^{\left(a\right)}$, with a dependency on the rate of deformation.

(4) $${\dot{\gamma }}^{\left(a\right)}={\dot{b}}^{\left(a\right)}f^{\left(a\right)}(\tau^{\left(a\right)}/g^{\left(a\right)})$$

(5) $$f^{\left(a\right)}(x)=x{\left|x\right|}^{n-1}$$

In the context of the reference shearing rate on the α slip system denoted by ${\dot{b}}^{\left(a\right)}$, the current strength of the α slip system represented by $g^{\left(a\right)}$, and the rate sensitivity exponent denoted by ‘n’, the value of $g^{\left(a\right)}$ can be expressed as follows:

(6) $${\dot{g}}^{\left(a\right)}=h_{\alpha \beta }{\dot{\gamma }}^{\left(\beta \right)}$$

where

(7) $$h_{\alpha \alpha }=h(\gamma )=h_{0}{\mathrm{sech}}^{2}\left|\frac{h_{0}\gamma }{\tau_{s}-\tau_{0}}\right|$$

(8) $$\gamma =\sum_{\alpha }\int_{0}^{t}\left|{\dot{\gamma }}^{\left(\alpha \right)}\right|dt$$

(9) $$h_{\alpha \beta }=qh(\gamma )\;\;\;\;\;{\;}_{\left(\alpha \neq \beta \right)}$$

In the equation provided, $h_{\alpha \beta }$ represents the latent hardening modulus, while *q* denotes the latent hardening constant. For a detailed and in-depth review of the crystal plasticity theory, refer to the work of Huang [60]. Wei et al. [39] investigated the effects of primary and secondary crystal orientations on the cyclic plastic deformation behavior of a recrystallized SC Ni-based superalloy using a crystal plasticity model and crystal plasticity finite element simulation. The results show that the maximum accumulated plastic shear strain of the SRX-SC system increases with increasing primary orientation, consistent with our research results.

**Figure 7:**
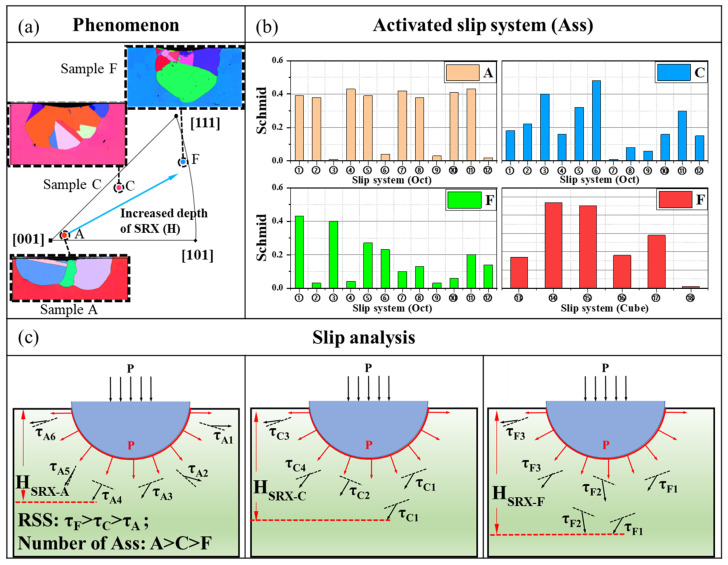
Schematic diagram of the effect of orientation on the depth of SRX.

Figure 7 shows the mechanism diagram of the SRX depths of A, C, and F samples after indentation with the change in crystal orientation. Specifically, Figure 7a delineates the precise crystal orientation positions of the three samples on the inverse pole figure. It can be observed in the figure that the smaller the angle of β, the closer it is to the [001] orientation in the inverse pole figure. As β increases, the position of the samples in the inverse pole figure gradually shifts away from the [001] orientation toward the [111] orientation (sample C and sample F in Figure 7a). Based on the analysis of the crystal slip theory and the activation mode of slip systems mentioned above, it can be concluded that sample A initiated multiple different slip systems with Schmid factors greater than 0.2 (Figure 7b) below the indentation (which are octahedral slip systems with different directions and sizes). The interaction of multiple slip systems makes the deformation more uniform, and the interaction of different slip faces can also make it difficult for the deformation of the slip face to expand downward, resulting in a smaller shear stress component below the indentation and a shallower SRX depth. Although the number of slip systems activated on both sides of the indentation is relatively small, the direction of the shear stress component of the slip system is essentially the same. This effectively promotes deformation on both sides of the indentation, leading to more SRXs on both sides of the indentation (Figure 3a and Figure 4a).

When orientation angle β increased to 27.8°, the number of octahedral slip systems activated near the indentation of sample C with a Schmid factor greater than 0.2 was less than that of sample A with a β angle of 4.7° (Figure 7b). But, the slip system’s direction below the indentation is essentially the same (Figure 6b), and multiple ⑥ slip systems with high Schmid factors are activated. This system exhibits a greater shear stress component along the indentation direction. Consequently, sample C, with an orientation angle of β to 27.8°, shows a deeper recrystallization depth beneath the indentation compared to sample A with a smaller β angle (Figure 7b,c). When the value of β increased to 48.4°, the number of octahedral slip systems with Schmid factors greater than 0.2 activated near the indentation of sample C decreased further (Figure 7b). Multiple Schmid factors with larger cubic slip systems were activated below the indentation, causing a further increase in the shear stress component along the indentation direction (Figure 6c and Figure 7b). This led to a more severe deformation along the indentation direction and, consequently, a greater depth of the recrystallized layer along the indentation direction (Figure 3).

### 3.2. Effects of External Stress on Recrystallization Behavior

**Figure 8:**
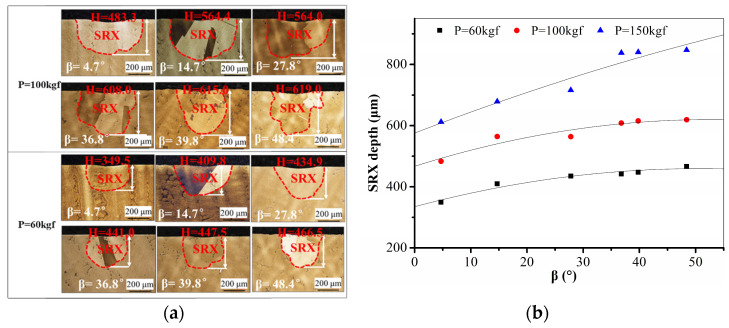
OM maps of SRX microstructure below the indentation at the loads of (**a**) 100 kgf and 60 kgf. (**b**) Statistical plot for the variation in SRX depth with crystal orientations at different external stresses.

Figure 8a depicts the SRX morphology in the vicinity of the indentation on the longitudinal section of specimens with varying crystal orientations following exposure to loads of 60 kgf and 100 kgf. The findings indicate that SRX takes place at the indentation subsequent to both 60 kgf and 100 kgf loading; however, the extent and profundity of SRX are notably smaller compared to those following a 150 kgf indentation. Specifically, the SRX depths at the indentation subsequent to a 60 kgf load ranged from 349.5 to 466.5 μm, while the SRX depths following a 100 kgf load ranged from 483.3 to 619.0 μm. The SRX depths subsequent to indentations at 150 kgf, 100 kgf, and 60 kgf were quantified and graphed to demonstrate the variation in SRX depth with crystal orientation (Figure 8b). The statistical analysis reveals that the SRX depth increases with an increase in β and tends to stabilize when β is equal to or greater than 36.8°.

For samples subjected to the same load, the depth of SRX increases with the increase in β. Furthermore, the depth of SRX increases in accordance with the load on the sample with the same crystal orientation. The amplitude of SRX depth also increases with higher values of β as the load increases. The larger the load, the greater the increase in SRX depth with the value of β. The primary reason for this is that an increased load causes significant plastic deformation and high deformation energy, directly leading to an increased degree of SRX. Notably, the evolution of SRX grain growth is more complex owing to the synergistic effect of the crystal orientation and external stress.

### 3.3. Growth Behavior of SRX in Dendrite and Interdendritic Regions

The deformation behavior of the SC superalloy strongly depends on external stress [18,61]. Notably, the center of the indentation was located in the interdendritic region, while the maximum SRX was observed on both sides of the indentation center (Figure 3a). In this regard, the microstructures and compositions of the relevant samples were analyzed using SEM. In general, the Ni-based superalloy consists primarily of two phases: the matrix phase, γ, and the strengthening phase, γ′. The γ phase exhibits a relatively soft and disordered face-centered cubic (FCC) structure, while the γ′ phase is characterized by a harder and ordered L1_2_ structure precipitate phase. The lattices of the γ and γ′ phases are aligned on the {100} plane, with crystallographic orientation relations of {100} γ // {100} γ′, <100>γ // <100>γ′. Both phases share similar electron diffraction patterns and atomic models. Detailed descriptions of the two-phase structures, including high-angle annular dark-field images (STEM-HAADF), electron diffraction, and atomic models, can be found in the literature [62].

**Figure 9:**
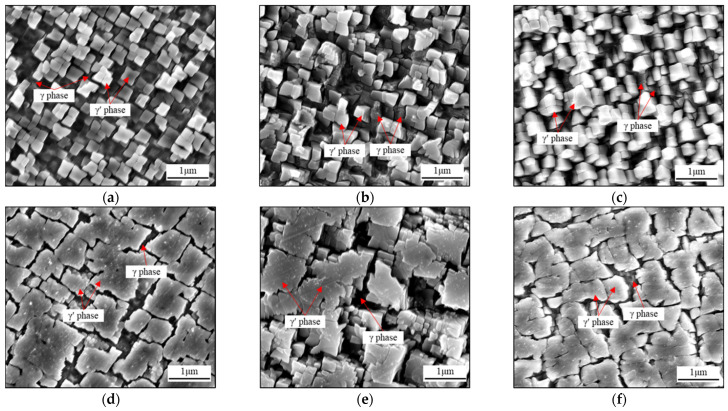
SEM maps of the as-cast γ′ phase morphology in dendrite core regions with β values of (**a**) 4.7°, (**b**) 27.8°, and (**c**) 48.4°, and interdendritic regions with β values of (**d**) 4.7°, (**e**) 27.8°, and (**f**) 48.4°.

Figure 9 shows the as-cast microstructure of the samples with β values of 4.7°, 27.8°, and 48.4°. The bright white square structure in the figure represents the γ′ phase, while the gray and dark colors between the γ′ phases represent the γ matrix phase. The γ′ phases are fine and uniformly distributed in the dendrite core regions (Figure 9a–c). Their morphologies are regular and are orderly stacked in the matrix. In the interdendritic regions (Figure 9d–f), the coarse γ′ phases with different shapes are disorderly distributed in the γ matrix. When the value of β is 4.7°, the average small cubic γ′ phase in the dendrite core regions is 0.17 μm in radius, and the volume fraction is approximately 70.6% (Figure 9a). The large irregular γ′ phase is observed in the interdendritic region, possessing a radius of 0.46 μm, and the volume fraction of this phase at the surface is 86.8% (Figure 9d). A similar phenomenon can be observed when the values of β are 27.8° and 48.4°. The γ′ phases in the dendrite core regions are smaller than those in interdendritic regions. In Figure 9a, the crystal orientation shows little deviation from the principal stress axis, and the square γ′ phases are observed. With an increased β, the inclined γ′ phases exhibit a three-dimensional cubic morphology, as shown in Figure 9b–c.

According to Nes et al. [63,64], the pinning force exerted by these coherent particles can be described as follows:

(10) $$F_{p}=\frac{2\pi AkTr^{2}}{3V}ln(\frac{c_{0}}{c_{eq}})-2\pi \gamma r$$

where *A* is Avogadro’s number, *k* is Boltzmann’s constant, *V* is the molar volume of the precipitate phase, *C*_0_ is solute concentration, *C_eq_* is equilibrium concentration, and *γ* is the energy of the coherent interface. Then, the pinning force exerted by the *γ′* phase in interdendritic regions (*F_pI_*) and dendrite core regions (*F_pD_*) can be described as follows:

(11) $$F_{pI}=\frac{2\pi AkTr_{I}^{2}}{3V}ln(\frac{c_{0}}{c_{eq}})-2\pi \gamma r_{I}$$

(12) $$F_{pD}=\frac{2\pi AkTr_{D}^{2}}{3V}ln(\frac{c_{0}}{c_{eq}})-2\pi \gamma r_{D}$$

To simplify the calculation, the values of *V*, *C*_0_, and *C_eq_* were assumed to be the same in the dendrite core and interdendritic regions. Then,

(13) $$F_{pI}=\frac{r_{I}^{2}}{r_{D}^{2}}F_{pD}+\frac{r_{I}(r_{I}-r_{D})}{r_{D}}f$$

where f=2πγ, $\gamma_{1}$ is the radius of the γ′ phase in interdendritic regions, and $\gamma_{D}$ is the radius of the γ′ phase in dendrite core regions. According to the above formula, it can be inferred that the pinning force exerted by γ′ phases in interdendritic regions is larger than that in dendrite core regions. For the sample with a β of 4.7°, the value of $r_{I}$ is 0.46 µm, while the value of $r_{D}$ is approximately 0.17 µm, i.e., $F_{I}\gg F_{D}$. This can be attributed to the faster development of SRX grains in the dendrite core regions than in the interdendritic regions.

**Figure 10:**
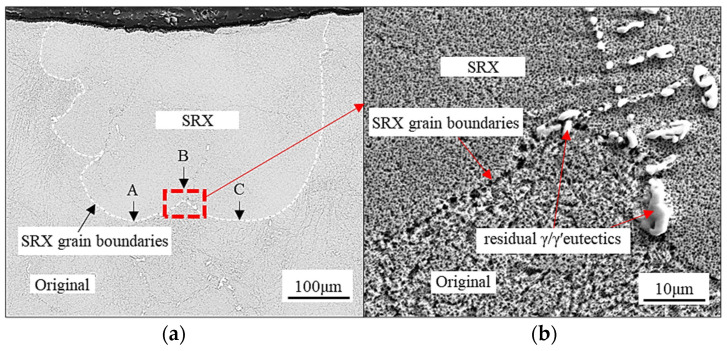
(**a**) SEM images of the SRX microstructures, and (**b**) localized magnified image of a recrystallized grain boundary.

In the interdendritic regions, besides the coarse γ′ phase, large γ/γ′ eutectics are observed. Therefore, a stronger pinning force appears at the SRX grain boundaries. Figure 10a shows a longitudinal section of the SRX microstructure below the indentation. The dotted line indicates the SRX grain boundary, and the local SRX grain boundaries in the dendrite regions are labeled with arrows A and C. The interdendritic regions are labeled with the arrow B. The region marked by B is located directly below the indentation center. However, the SRX grain boundaries are concave in the region marked by arrow B, indicating that the SRX grain boundaries are subjected to greater resistance than those in the regions marked by arrows A and C. In Figure 10b, the amplification image of the region marked by arrow B shows a cluster of residual γ/γ′ eutectics at the SRX grain boundary, which suggests that the residual γ/γ′ eutectics delay the appearance SRX grain boundary during the downward-advancement process.

Based on the experimental observations and microstructural analysis, it can be concluded that the pinning effect of SRX is greater in the interdendritic region than in the dendrite region. This is mainly due to the larger size of the γ′ phase in the interdendritic region, which is in agreement with the reports of previous research [65,66]. Additionally, the presence of the γ/γ′ eutectic in the interdendritic region is believed to be the more significant factor contributing to the coarse γ′ phase. Although the volume fraction of the γ/γ′ eutectic is smaller than that of the γ′ phase, its larger size results in a greater pinning effect. This can be seen in area B in Figure 10a, where the SRX grain is unable to penetrate the γ/γ′ eutectics in the growth process.

In the solidification process of SC castings, the elements Cr, Co, Mo, Re, and W are enriched in the dendrite core regions, while the γ′ phase-forming elements, such as Al, Ti, and Ta, are enriched within the interdendritic regions [41,67,68]. Due to the lack of γ′ phase-forming elements in the dendrite core regions, no γ/γ′ eutectic is formed during solidification, and the size of the γ′ phase is also small (Figure 9). The difference in microstructure between the dendrite and interdendritic regions leads to distinct SRX behavior. The cause of this difference lies in the varying microscopic compositions of these two regions.

The process of solution heat treatment can partially reduce dendrite segregation and promote microstructural homogenization; however, due to time constraints, it may not entirely eliminate compositional variations between dendrite and interdendritic regions. Consequently, a residual γ/γ′ eutectic may persist after solution heat treatment. Increasing the solution heat treatment temperature or prolonging the heat treatment time is beneficial for eliminating the residual γ/γ′ eutectic. However, it will also increase the risk of incipient melting and SRX. To enhance the quality of SC superalloy castings, it is advisable to optimize the selection of crystallographic orientations or employ the seed crystal technique to minimize deviations in crystal orientation. Furthermore, precautions should be taken to prevent localized plastic deformation of SC superalloy castings during shell removal and transportation procedures.

This paper delineates the variations in slip system activation patterns and quantities subsequent to room temperature deformation of SC samples. It also elucidates the SRX characteristics and deformation mechanism of samples oriented at different angles relative to the [001] orientation. Furthermore, it discusses the inhibitory impact of eutectic and coarse γ′ phases on SRX through the lens of element segregation and the size of the γ′ phase. Moreover, it expounds on the distinct SRX growth behaviors observed in dendritic and interdendritic regions. The findings of this study can offer valuable insights into effectively managing SRX in the manufacturing process of SC castings.

## 4. Conclusions

DD5 Ni-based SC rods with different crystal orientation deviations were subjected to indentation loads. The relevant deformation and SRX mechanisms were investigated in detail. The following conclusions were drawn:

(1) The SRX behavior of the DD5 Ni-based SC rod exhibits a correlation with orientation, where the depth of SRX increases with higher β values. In SC superalloys, room temperature deformation is primarily driven by crystal slips. The activation of slip systems is dependent on crystal orientation, leading to the activation of different slip systems based on the β value.
(2) Load on sample affects the depth of SRX, with higher loads resulting in deeper SRX layers.
(3) Variations in element distribution and the size disparity of the γ′ phase result in the formation of γ/γ′ eutectics and larger γ′ phases in DD5 castings. Residual γ/γ′ eutectics and coarse γ′ phases serve as impediments to the growth of SRX during heat treatment, thereby impeding the migration of SRX grain boundaries.
(4) By optimizing the spiral crystal selection process or utilizing seed crystal technology to acquire SC castings with a smaller crystal orientation, and preventing local deformation caused by collisions during the removal of shells of SC castings, the occurrence of SRX in SC superalloys can be effectively controlled.

## Figures and Tables

**Table 1:** Nominal composition of the DD5 alloy [43].

Element	Cr	Co	W	Mo	Al	Ta	Hf	Re	Ti	Ni
wt. (%)	7.0	7.5	5.0	1.5	6.2	6.5	0.15	3.0	-	Bal.

**Table 2:** The crystal orientation deviation angle (β) of the investigated samples.

Sample	A	B	C	D	E	F
β (°)	4.7	14.7	27.8	36.8	39.8	48.4

**Table 3:** Schmid factors for the octahedral slip systems in the samples with β values of 4.7°, 27.8°, and 48.4°.

Slip Plane	$\left(1\bar{1}1\right)$			$\left(\bar{1}11\right)$			$\left(\bar{1}\bar{1}\bar{1}\right)$			$\left(11\bar{1}\right)$		
Slip Direction	$\left\langle \bar{1}01\right\rangle$	$\left\langle \bar{1}\bar{1}0\right\rangle$	$\left\langle 011\right\rangle$	$\left\langle \bar{1}\bar{1}0\right\rangle$	$\left\langle \bar{1}0\bar{1}\right\rangle$	$\left\langle 0\bar{1}1\right\rangle$	$\left\langle \bar{1}01\right\rangle$	$\left\langle \bar{1}10\right\rangle$	$\left\langle 0\bar{1}1\right\rangle$	$\left\langle \bar{1}0\bar{1}\right\rangle$	$\left\langle \bar{1}10\right\rangle$	$\left\langle 011\right\rangle$
Slip System No.	①	②	③	④	⑤	⑥	⑦	⑧	⑨	⑩	⑪	⑫
Sample A (4.7°)	0.39	0.38	0.01	0.43	0.39	0.04	0.42	0.38	0.03	0.41	0.43	0.02
Sample C (27.8°)	0.18	0.22	0.4	0.16	0.32	0.48	0.01	0.08	0.06	0.16	0.30	0.15
Sample F (48.4°)	0.43	0.03	0.4	0.04	0.27	0.23	0.10	0.13	0.03	0.06	0.2	0.14

**Table 4:** Schmid factors of cubic slip systems in the sample with a β of 48.4°.

Slip Plane	$\left(100\right)$		$\left(010\right)$		$\left(001\right)$	
Slip Direction	$\left\langle 01\bar{1}\right\rangle$	$\left\langle 011\right\rangle$	$\left\langle \bar{1}01\right\rangle$	$\left\langle \bar{1}0\bar{1}\right\rangle$	$\left\langle \bar{1}10\right\rangle$	$\left\langle \bar{1}\bar{1}0\right\rangle$
Slip System No.	⑬	⑭	⑮	⑯	⑰	⑱
Sample F (48.4°)	0.17	0.47	0.45	0.18	0.29	0.01

## Data Availability

Data are contained within the article.
